# Kinetic ELISA in Microfluidic Channels

**DOI:** 10.3390/bios1020058

**Published:** 2011-06-17

**Authors:** Naoki Yanagisawa, Debashis Dutta

**Affiliations:** Department of Chemistry, University of Wyoming, Laramie, WY 82071, USA; E-Mail: nyanagis@uwyo.edu

**Keywords:** microfluidic, ELISA, Blue Tongue Virus, Epizootic Hemorrhagic Disease Virus, viral antibodies, fluorescence

## Abstract

In this article, we describe the kinetic ELISA of Blue Tongue and Epizootic Hemorrhagic Disease viral antibodies in microfluidic channels by monitoring the rate of generation of the enzyme reaction product under static conditions. It has been shown that this format of the immunoassay allows very reliable quantitation of the target species using inexpensive glass microchips and a standard epifluorescence microscope system coupled to a CCD camera. For the viral antibodies assayed here, the limit of detection (LOD) for the analyte concentration in our microchips was established to be 3–5 times lower than that obtained on commercial microwell plates using a fiftieth of the sample volume and less than a third of the incubation time. Our analyses further show that when compared to the end-point ELISA format, the kinetic mode of this assay yields an improvement in the LOD by over an order of magnitude in microfluidic devices. This benefit is primarily realized as the observed variation in the background fluorescence (signal at the start of the enzyme reaction period) was significantly larger than that in the rate of signal generation upon repeating these assays in different microchannels/microchips. Because the kinetic ELISA results depend only on the latter quantity, the noise level in them was substantially lower compared to that in its end-point counterpart in which the absolute fluorescence measurements are of greater significance. While a similar benefit was also recorded through implementation of kinetic ELISAs on the microwell platform, the improvement in LOD registered in that system was not as significant as was observed in the case of microfluidic assays.

## 1. Introduction

Determining trace quantities of proteins in complex biological mixtures is one of the most important objectives of bioanalytical chemistry. In order to detect the onset of a disease at an early stage for example, highly sensitive assays are desirable that can quantitatively estimate small amounts of antigens/antibodies circulating in bodily fluids. Immunoassays are very important tools in this regard because of their ability to identify and quantitate specific proteins based on the high affinity between an antigen and an antibody. Among the different types of immunoassays developed to date, Enzyme Linked Immunosorbent Assay or ELISA is arguably one of the most practiced ones. The key advantage of using ELISA methods is the inherent signal amplification in this technique which allows one to reliably quantitate very small amounts of proteins present in biological samples [1]. While the greater sensitivity of the ELISA method is impressive, it is nevertheless desirable to further improve the limit of detection for this technique extending its applicability to more challenging assays [2].

In an effort to reduce the sample volume requirement in the ELISA method, this immunoassay has been implemented on the microfluidic platform by several researchers in conjunction with different detection/quantitation techniques [3]. One of the simpler versions among these relies on the use of a non-fluorescent enzyme substrate that gets transformed into a detectable fluorescent product over the course of the assay [4]. The amount of this fluorescent reporter in most cases, however, has been either detected off-chip or in the analysis channel after a fixed period of enzyme reaction, *i.e.*, the end-point ELISA format [5,6]. In this article, we report a substantial improvement in both the reproducibility and the limit of detection of the fluorescence based microfluidic ELISA method using the kinetic format of the assay. Antibodies to the Blue Tongue Virus (BTV) and Epizootic Hemorrhagic Disease Virus (EHDV) grown using hybridoma cell lines and previously employed to the detection of these viral infections in a variety of ruminants [7,8,9] were used as the analytes for evaluating the performance of these assays. Both BTV and EHDV are arthropod-borne pathogenic species that cause serious illness in livestock such as sheep, goats, cattle and deer [10,11]. There is no efficient treatment currently available for these viral infections and therefore their detection at an early stage is often key to preventing their outbreak among artiodactyles in zoos and livestock farms for minimizing economic damages. Interestingly, both of these viruses are known to be transmitted by biting midges (*Culicoides* species) and their infections tend to be confounding due to antigenic similarity [12]. As a result, there is a need for developing assays that would allow the specific detection of each these conditions in a time and cost effective manner. To this end, several ELISA methods have been successfully developed [7,8,9] whose utility in point-of-care diagnostic applications can be significantly improved through their implementation on portable platforms. In this work, we demonstrate a microfluidic ELISA for quantitatively determining the levels of BTV (serotype 11) and EHDV (serotype 2) antibodies with a higher sensitivity than currently possible on commercial microwell plates. Our experiments show that when the concentrations of these analytes in the microfluidic assays are arrived at by comparing the rate of signal generation rather than the signal itself, the noise in the system is substantially reduced. This leads to a more reliable quantitation particularly under conditions when the change in signal over the enzyme reaction period is small compared to the background fluorescence (signal at the start of the enzyme reaction) in the system as is the case here. While this result was observed to be valid even in the case of microwell plates, the benefit of employing the kinetic format of the assay over its end-point version in microwell based ELISAs was found to be not as significant.

## 2. Materials and Methods

### 2.1. Microchip Design

**Figure 1:**
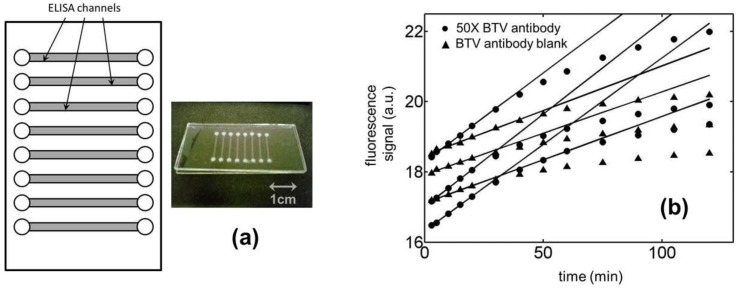
(**a**) Microchip design used in the present study. (**b**) Temporal variation in the fluorescence signal in microchannels with an active ELISA surface for 50× BTV antibody samples and its corresponding blanks. The presented data set are for 3 repetitions of each of these ELISAs in separate fluidic ducts. Although in this particular case, all of the concerned microchannels were located on the same microchip, the assay-to-assay variability in our experiments was dominated by the fluctuations in the channel-to-channel characteristics. The observed chip-to-chip variability was not any different from the channel-to-channel variability in our system.

For fabricating the microfluidic devices employed in this work, bottom substrates and cover plates made from borosilicate glass were purchased from Telic Company (Valencia, CA). While the purchased cover plates had both their faces unprotected, the bottom substrates came with a thin layer of chromium and photoresist laid down on one of their surfaces. The fabrication process for the microchips was initiated by photolithographically patterning [13] the desired channel layout (see Figure 1(a)) on the bottom substrate using a custom designed photomask created through Fineline Imaging Inc. (Colorado Springs, CO). The length and the width of our analysis channels were chosen to be 1.5 cm and 500 µm, respectively, allowing us to accommodate 8 fluidic conduits in each of the 2” × 1” microchips. After completion of the photopatterning process, the photoresist layer was cured in microposit developer MF-319 (Rohm and Haas) and the chromium layer removed along the channel network with a chromium etchant (Transene Inc.). The channels were then etched to a chosen depth of 30 µm using a solution of buffered oxide etchant purchased from Transene Inc. Access holes were punched into the glass plate at the channel terminals using a micro-abrasive particle blasting system (Vaniman Manufacturing Company) for introducing the ELISA reagents. Finally, the microfluidic network was sealed off by bringing a cover plate in contact with the bottom substrate in de-ionized water and then allowing the two plates to bond under ambient conditions overnight [14]. No external fluidic ports were attached to the access holes of our device in order to minimize the volume of ELISA reagents needed to derivatize the glass microchannels. The evaporation of chemicals during the incubation steps was prevented in this situation by sealing the access holes with adhesive tapes. All reagents were introduced and purged from the microchannels through the use of an in-house vacuum supply. The device thus fabricated was prepared for an experiment by first rinsing its conduits with 1 N sodium hydroxide (Sigma-Aldrich) for 1 h and then with de-ionized water for 10 min. The channels were later dried at 80 °C in a forced-air convection oven before treating them with a solution of (3-aminopropyl)triethoxysilane (Sigma-Aldrich) for an hour under ambient conditions. Subsequently, the fluidic network was rinsed with methanol (Fisher Scientific) and then reacted at room temperature with an aqueous solution containing 5% w/v glutaraldehyde (Sigma-Aldrich) for another 60 min to create a surface that could be covalently attached to the amine groups on a protein molecule. 

### 2.2. ELISA Reagents

Phosphate (pH 7.4) and carbonate (pH 9.4) buffers utilized in the reported immunoassays were prepared by dissolving appropriate amounts of sodium carbonate (J.T. Baker), bicarbonate (EMD Chemicals)/monophosphate (Fisher Scientific), biphosphate (Fisher Scientific) in de-ionized water (DirectQ Water Purification System, Millipore). The concentration of both these buffers was chosen to be 0.1 M in all our experiments. The analytes quantitated in this study include antibodies to the BTV and EHDV antigens. The mother solutions for these antibodies were the supernatant fluids from hybridoma cultures prepared using the spleens of adult female BALB/c mice that had been immunized by a series of intraperitoneal and intravenous inoculations with recombinant BTV and EHDV particles, respectively [8,15]. The polyclonal capture antibodies for the assays were produced by inoculation of rabbits with recombinant BTV/EHDV and drawing 10 mL of their blood two weeks after infection. This source liquid was then centrifuged at 1,000× g for 15 min to obtain the mother solution for the capture species. The recombinant viral antigens were expressed in Sf9 insect cells by infection with recombinant baculovirus containing the cloned VP7 gene of BTV/EHDV using the procedure described by Mecham and Wilson [8]. The mother solutions for these antigens used in our study were the supernatant fluids from their respective Sf9 cell cultures. Biotinylated goat anti-mouse immunoglobulin in combination with streptavidin horseradish peroxidase conjugate (both purchased from BioGenex Laboratories) were used for detecting the BTV/EHDV antibodies immobilized on the ELISA surface. The enzyme substrate chosen for our assays was a mixture of Amplex red (Invitrogen) and hydrogen peroxide (Avantor Performance Materials) which generated resorufin as the detectable reaction product.

### 2.3. Preparation of the ELISA Surface

The ELISA surface in the glutaraldehyde coated microchannels was initiated by incubating a solution containing excess amounts of polyclonal BTV/EHDV capture antibody prepared in carbonate buffer for 1 h. Preliminary experiments showed (data not presented) that the concentration of these capture species did not affect the assay results provided a 100-fold (100×) or lesser dilution of it was used during the incubation step. In this situation, a 50× dilution of this source liquid was chosen to ensure the excess of the capture species on the ELISA surface. After having immobilized the capture antibodies, the fluidic conduits were incubated with a solution containing 0.1 M lysine (Sigma-Aldrich) in carbonate buffer for 30 min and subsequently treated with a 1% (w/v) BSA solution (Sigma-Aldrich) in the same background electrolyte for 1 h to block off the unreacted sites on the channel surface. At this point, depending on the kind of analyte chosen for our assay, the analysis channels were exposed to a solution containing excess amounts of recombinant BTV or EHDV particles prepared in the phosphate buffer for 1 h. During this coating step, a 10× dilution of the mother solution for the BTV/EHDV antigen was used to ensure its excess relative to the analyte (viral antibodies) on the ELISA surface. This dilution factor was again arrived at through preliminary experiments (data not shown) which established that the 10× antigen concentration was not limiting the ELISA signal in our system. Subsequently, the microchannels were reacted for an hour with different concentrations (6.25–200× dilutions of their respective mother solutions) of the viral antibody analytes diluted in phosphate buffer to establish their limits of detection. Following this immobilization, excess amounts of biotinylated goat anti-mouse immunoglobulin (10× dilution of a mother solution obtained from BioGenex Laboratories) prepared in phosphate buffer was introduced into the entire microfluidic network and incubated for 1 h. Finally, the microchannels were treated with excess amounts of streptavidin-horseradish peroxidase conjugate (10× dilution of a mother solution obtained from BioGenex Laboratories) prepared in phosphate buffer containing 0.05% v/v Tween20 (Sigma-Aldrich) for 1 h to complete the ELISA surface.

### 2.4. Microfluidic Device Operation

The enzyme-linked immunosorbent assays were realized in our microfluidic devices by introducing a solution containing 10 µM Amplex Red and 5 µM hydrogen peroxide in phosphate buffer into the analysis conduits with an active ELISA surface. The enzyme reaction was performed in our assays by maintaining an air temperature of 37 °C around the microchip (measured using a thermometer) through placement of a heating fan close to it. Because the optical detection in our set-up could be made without disturbing the air flow around the microchip in any significant way (except for minor movements of the automated microscope stage when scanning the different microchannels) no temperature fluctuations were recorded in the thermometer reading during the data collection process. It must be noted that without this temperature control, the assay-to-assay variability in the ELISA signal was observed to significantly larger in our experiments due to fluctuations in the ambient room temperature. Fluorescence measurements at the center of each ELISA channel were made using an epifluorescence microscope (Nikon) with band-pass excitation (528–543 nm) and emission (590–650 nm) optical filters. The fluorescence image thus obtained was recorded using a CCD camera (Roper Scientific) and analyzed with the Adobe Photoshop software. To minimize any unwanted signal generation in our system through photo-oxidation of Amplex Red [16], the ELISA regions were exposed to the excitation beam for only 1 s during the imaging process through the use of a mechanical shutter. This exposure time was arrived at through preliminary experimentation which established no noticeable drift in signal due to photo-oxidation effects upon exposure of the ELISA region to the excitation beam for less than 3 s. Moreover, the cumulative influence of repeated beam exposures was assessed by making as many as 10 measurements over a 30 min ELISA reaction period which again showed no detectable change in the fluorescence data due to either non-enzymatic conversion of Amplex red or heat generation by the excitation beam. In our study, the beam size was further controlled such that it illuminated a circular region about a millimeter in diameter around the center of the concerned analysis channel. During this detection process the metallic microscope stage masked the other microchannels in the device minimizing their exposure to the excitation beam. The camera exposure time was chosen to be 100 ms in all our measurements.

### 2.5. Microwell Plate ELISA

While a majority of the steps in preparing the microwell plates (Immulon II, Dynatech Laboratories) for an ELISA reaction was identical to those employed in the microfluidic version of the assay, there were three important distinctions between the two cases. Firstly, the BTV and EHDV capture antibodies were laid down on the surface of the microwell plates without the use of any linkers such as (3-aminopropyl)triethoxysilane and glutaraldehyde as was employed in the glass microchannels. Secondly, the incubation periods for these capture antibodies necessary for generating substantial ELISA signal in the microwells was found to be 24 h as compared to 60 min for the corresponding procedure in the microfluidic ducts. Note that this incubation process was carried out at 4 °C in the former case while implemented at room temperature in the latter. The incubation periods and conditions for the remaining biological reagents were chosen to be identical for both the microchip and microwell based ELISAs in our experiments. Overall, this corresponded to about 30 h of device preparation time for the microwell plates as opposed to only 10 h for the fluidic microchips. It must be pointed out that this saving in the device preparation time did not translate into any reduction in the labor involved in performing the assays on the microchips. Finally, it is also to be noted that the volume of biological reagents used in all of the incubation steps for the microwell based ELISAs was 100 µL as compared to 2 µL in the case of the microfluidic assays. 

## 3. Results and Discussion

Because a major objective of our research was to compare the sensitivity of the kinetic ELISAs to their end-point versions, it was necessary for us to ensure that the enzyme reaction followed a zeroth order kinetics during the course of the assay. To this end, a series of experiments were performed to establish a suitable enzyme reaction period over which the fluorescence in the microchannels/microwells increased linearly with time. In this regard, we have presented kinetic data from six different ELISA microchannels (see Figure 1(b)) three of which were incubated with a 50× BTV antibody solution and the other three were blanks for the same analyte. Although all of the concerned microchannels were located on the same microchip in this case, the variability in the observed ELISA signal across them was very similar to those noticed among analysis channels etched on different microchips. In other words, the assay-to-assay variability in our experiments was dominated by the fluctuations in the channel-to-channel characteristics and not by the differences in the microchips. No ELISA channel was used twice for an assay in our study. As may be seen from Figure 1(b), the fluorescence in these fluidic conduits grew linearly in time with coefficients of determination (R^2^ value) greater than 0.97 during the first 30 min of the assay. There onwards the rate of signal generation was observed to slow down deviating from the desirable zeroth order enzyme kinetics [17]. In this situation, the ELISA reaction period was chosen to be 30 min in all our experiments. Figure 1(b) also shows that when the same assay is repeated in different microchannels/microchips the variation in the background fluorescence (signal at time = 0) in them was significant compared to the change in signal over the 30 min enzyme reaction period. Interestingly, the rate of signal generation in all these cases was observed to be very reproducible for both the 50× BTV-antibody sample and its corresponding blank. In the case of the 50× BTV-antibody sample for example, the mean value for the background fluorescence across five different ELISA microchannels was measured to be 17.29 ± 0.98 a.u. (5.67% RSD) while the corresponding number for the rate of signal generation in the same conduits was determined to be 0.0511 ± 0.0018 a.u./min (3.52% RSD). In this situation, the average value (using 5 measurements) for the change in fluorescence signal over the 30 min enzyme reaction period in these assays was 1.53 a.u. which was comparable to the standard deviation in the background fluorescence across the different ELISA channels, *i.e*., 0.98 a.u. Notice that because the signal and noise level in end-point ELISAs are proportional to these quantities respectively, their similar magnitude implied a lower reliability of the experimental data for this assay format. On the other hand, the small relative standard deviation in the rate of ELISA signal generation, *i.e*., 3.52%, suggests that it may be possible to obtain significantly lower limits of detection for the target analyte using the kinetic version of the assay.

**Figure 2:**
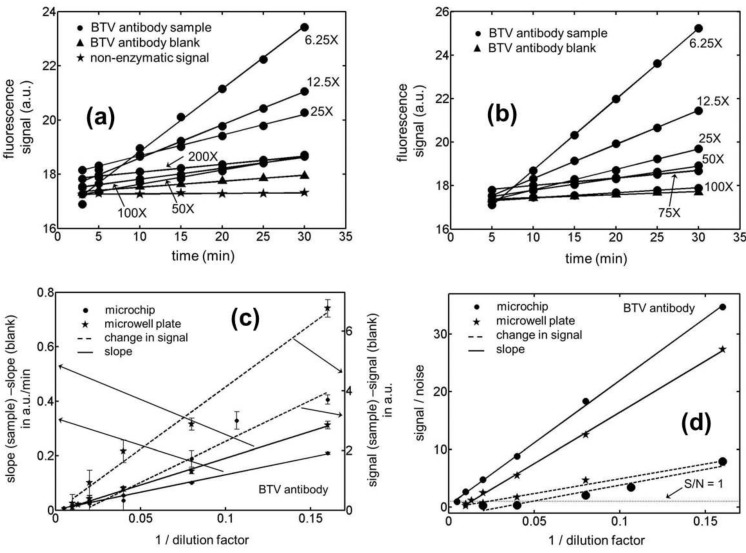
Temporal variation in the fluorescence signal for Blue Tongue Virus (BTV) antibody samples in (**a**) microchannels (**b**) microwells with an active ELISA surface. (**c**) Calibration curves for the kinetic and end-point ELISAs in microchips and microwell plates for BTV antibody samples. Note that while the rate of signal generation for a sample minus that for its corresponding blank (left axis) has been plotted for quantitating the kinetic ELISAs, the absolute change in signal over a fixed enzyme reaction period minus that for its corresponding blank (right axis) was used as a measure for quantitating the end-point immunoassays. (**d**) Limit of detection (LOD) curves for the kinetic and end-point ELISAs in microchannels and microwells for the BTV antibody samples. The noise level (N) in these calculations was evaluated as 3-times the standard deviation in the signal (S). The LOD for an assay was estimated as the dilution factor for which S/N = 1.

Having established a suitable enzyme reaction period for our assays, fluorescence measurements were made in microchannels/microwells that were incubated with different concentrations of the BTV antibody (see Figure 2(a,b)). The data shows a fairly linear change in this signal with time for both the microfluidic and microwell based assays. There is however, again a substantial variation in the background fluorescence in going from one assay compartment to the other particularly in the case of the microchannel based ELISAs. As a result, the fluorescence curves for the different sample concentrations are seen to cross each other over the assay period, more frequently for the microchips than for the microwell plates. In Figure 2(c), we have compared the signal generated by the sample minus the corresponding blank for the end-point and kinetic mode of these assays on both the microchip and microwell platforms. Note that while only one fluorescence measurement was made after a fixed enzyme reaction period in the end-point ELISAs, 6–7 readings were taken over the same duration in the kinetic version of the assay. This enzyme reaction period was chosen to be 30 min in all our experiments which was arrived at based on the results presented in Figure 1(b). The data presented in this figure suggest that if this reaction time is chosen to be less than 15 min or greater than half-an-hour, the signal to noise ratio in the system is compromised. For the choice of a 30 min enzyme reaction period, the measured signal for both the ELISA formats was observed to vary linearly with the inverse of the dilution factor for the BTV-antibody sample. Figure 2(c) however shows a significantly larger noise level (measured in terms of the standard deviation for the signal) in the end-point ELISAs which arises due to a substantial variation in the background fluorescence (signal at the start of the enzyme reaction period) going from one assay compartment to the other. Moreover, the disparity in the noise levels for the two ELISA formats was also observed to be substantially greater in the microchannels compared to that in the microwells. While the reasons behind this observation are not completely clear, it is possible that the larger surface roughness in the chemically etched fluidic ducts may have resulted in a larger variability in the quality of the surface coatings within them. In addition, it was also qualitatively observed that the amount of light scattered by the rougher microchannels was greater than that by the microwell plates. 

**Figure 3:**
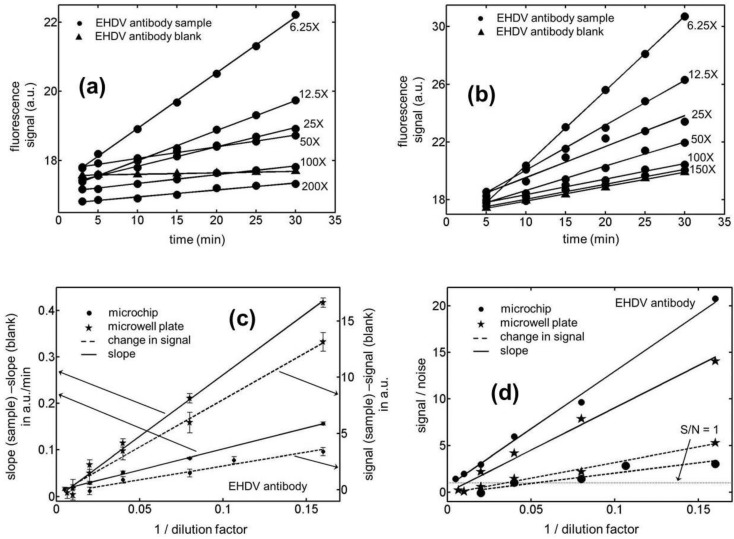
Temporal variation in the fluorescence signal for Epizootic Hemorrhagic Disease Virus (EHDV) antibody samples in (**a**) microchannels (**b**) microwells with an active ELISA surface. (**c**) Calibration curves for the kinetic and end-point ELISAs in microchips and microwell plates for EHDV antibody samples. (**d**) Limit of detection (LOD) curves for the kinetic and end-point ELISAs in microchannels and microwells for the EHDV antibody samples. Notice that the *x*-coordinate in (c) and (d) has been chosen to be the reciprocal of dilution factor rather than the dilution factor itself. Our analyses show that if the latter choice is made, the linear variation between the *x* and *y* data points is compromised.

In Figure 2(d), we have presented the signal to noise ratio (S/N) in our BTV antibody ELISA experiments as a function of the sample dilution factor. The noise level in these evaluations was assumed to be three-times the standard deviation in the magnitude of the signal which establishes the limit of detection for these curves to be the dilution factor when S/N = 1. For all of the measurements presented in this figure, the noise level was estimated based on five independent experiments both for the end-point and kinetic assays. Based on these measurements, the limits of detection (in terms of dilution factors) were established to be 420.1 ± 7.9 and 21.1 ± 4.4 for the kinetic and end-point ELISAs, respectively, in the microfluidic channels. The 95% confidence intervals for these LOD values are estimated to be 420.1 ± 9.8 and 21.1 ± 5.5, respectively, based on the two-sided *t*-distribution. This corresponds to a nearly 20-fold reduction in this quantity on adopting the kinetic version of the assay over its end-point format in microchips. Moreover, the data shows a significantly greater reproducibility in the former mode of the immunoassay yielding a relative standard deviation of 1.9% in the limit of detection (LOD) as compared to 20.9% for the latter case. For the microwell based assays, the LODs for the kinetic and end-point ELISAs were estimated to be 142.3 ± 13.4 and 44.2 ± 4.1, respectively. In this situation, the 95% confidence intervals for these quantities turn out to be 142.3 ± 16.7 and 44.2 ± 5.1, respectively, based on the two-sided *t*-distribution. This corresponds to about a 3-fold improvement in the smallest concentration that could be reliably quantitated and the relative uncertainly in this figure of merit on adopting the kinetic version of the assay over its end-point format in microwells. On comparing the kinetic ELISAs in microchannels to those in microwells, the former platform has been shown to yield a 3-fold lower LOD with at least 5-fold higher precision. But for the end-point immunoassays the trend reverses and the latter platform was observed to perform twice as well as the microfluidic devices. For the EHDV antibodies, similar trends to those reported above were again noticed for the immunoassays (see Figure 3). In the case of the microfluidic experiments, the LOD in terms of the dilution factors were estimated to be 307.3 ± 5.9 and 18.8 ± 6.3, respectively, for the kinetic and end-point version of the ELISAs. The corresponding numbers for the microwell plates were 63.1 ± 17.2 and 29.3 ± 8.1, respectively. Based on the two-sided *t*-distribution, the 95% confidence intervals for these four LODs can be shown to be 307.3 ± 7.3, 18.8 ± 7.8, 63.1 ± 21.4 and 29.3 ± 10.1, respectively. Therefore, the improvement in LOD yielded by the microchips over the microwell plates was a factor of 4.9 for kinetic EHDV antibody ELISAs while the latter platform performed only about 1.5 fold better than the microchips in the case of the end-point assays. As before, the best reproducibility was obtained for the microchip based kinetic ELISAs with relative standard deviation of 1.9%.

## 4. Conclusions

The present work demonstrates a significantly higher detection sensitivity and reproducibility for the kinetic version of microfluidic ELISAs in assessing BTV and EHDV antibodies. This format of the immunoassay has been shown to yield a 3–5 fold reduction in the limit of detection for the analytes of interest over that realized on microwell plates using a fiftieth of the sample and a third of the incubation time. Moreover, the experimental reproducibility for this version of ELISA (measured in terms of the relative standard deviation for the signal) was found to be at least 5-times better than the other formats of the assay investigated in this work. The lower LODs reported above was primarily realized as a result of the larger reproducibility and hence a smaller noise level in the kinetic version of the microfluidic assays. On the other hand, the variation in the background fluorescence for microfluidic ELISAs performed in different microchannels/microchips was observed to be large in our experiments and yielded poor signal to noise ratios in the end-point assays. It must be also pointed out that the LODs reported in this work have been expressed in terms of the dilution factors for the mother solutions of the analytes in the absence of any standard BTV/EHDV antibody samples. While this fact makes our assays somewhat semi-quantitative, the results included in the current work should be readily applicable to quantitative ELISAs for which standard samples are available. It must be emphasized however that the range of dilution factors used for the recombinant BTV/EHDV antibody mother solutions in this study are comparable to that have been previously employed in competitive/block ELISAs for detecting BTV and EHDV infections in ruminant population [7,8,9]. In this situation, the reported limits of detection have relevance to detecting an outbreak of BTV/EHDV infections in zoos and livestock farms. In addition to the channel-to-channel/chip-to-chip reproducibility described above, we also performed assays using three different batches of the analyte antibodies that were prepared following the exact same procedure described previously [7,8,9]. Although similar trends were observed for both the kinetic and end-point assays using these batches of analyte, the actual values for the limits of detection varied by as much as 20%. This variation however, was relatively small compared to the improvement in LOD reported here using the kinetic format of microfluidic ELISAs. Finally, it must be pointed out that the assays developed in our current work are highly specific for assessing the levels of BTV and EHDV antibodies in bodily fluids in spite of the similarity between these antigens. This issue has been previously investigated by our collaborator, Dr. James Mecham, in a series of publications [7,8,9].
